# Feasibility of CT radiomics to predict treatment response of individual liver metastases in esophagogastric cancer patients

**DOI:** 10.1371/journal.pone.0207362

**Published:** 2018-11-15

**Authors:** Remy Klaassen, Ruben T. H. M. Larue, Banafsche Mearadji, Stephanie O. van der Woude, Jaap Stoker, Philippe Lambin, Hanneke W. M. van Laarhoven

**Affiliations:** 1 Amsterdam UMC, University of Amsterdam, Department of Medical Oncology, Cancer Center Amsterdam, Amsterdam, Netherlands; 2 Amsterdam UMC, University of Amsterdam, LEXOR, Laboratory for Experimental Oncology and Radiobiology, Cancer Center Amsterdam, Amsterdam, Netherlands; 3 The D-Lab: Decision Support for Precision Medicine, GROW-School for Oncology and Developmental Biology, Maastricht Comprehensive Cancer Centre, Maastricht University Medical Centre, Maastricht, The Netherlands; 4 Amsterdam UMC, University of Amsterdam, Department of Radiology and Nuclear Medicine, Cancer Center Amsterdam, Amsterdam, Netherlands; Institute of Automation Chinese Academy of Sciences, CHINA

## Abstract

In this study we investigate a CT radiomics approach to predict response to chemotherapy of individual liver metastases in patients with esophagogastric cancer (EGC). In eighteen patients with metastatic EGC treated with chemotherapy, all liver metastases were manually delineated in 3D on the pre-treatment and evaluation CT. From the pre-treatment CT scans 370 radiomics features were extracted per lesion. Random forest (RF) models were generated to discriminate partial responding (PR, >65% volume decrease, including 100% volume decrease), and complete remission (CR, only 100% volume decrease) lesions from other lesions. RF-models were build using a leave one out strategy where all lesions of a single patient were removed from the dataset and used as validation set for a model trained on the lesions of the remaining patients. This process was repeated for all patients, resulting in 18 trained models and one validation set for both the PR and CR datasets. Model performance was evaluated by receiver operating characteristics with corresponding area under the curve (AUC). In total 196 liver metastases were delineated on the pre-treatment CT, of which 99 (51%) lesions showed a decrease in size of more than 65% (PR). From the PR set a total of 47 (47% of RL, 24% of initial) lesions were no longer detected in CT scan 2 (CR). The RF-model for PR lesions showed an average training AUC of 0.79 (range: 0.74–0.83) and 0.65 (95% ci: 0.57–0.73) for the combined validation set. The RF-model for CR lesions had an average training AUC of 0.87 (range: 0.83–0.90) and 0.79 (95% ci 0.72–0.87) for the validation set. Our findings show that individual response of liver metastases varies greatly within and between patients. A CT radiomics approach shows potential in discriminating responding from non-responding liver metastases based on the pre-treatment CT scan, although further validation in an independent patient cohort is needed to validate these findings.

## Introduction

The outcome of esophageal and gastric carcinomas (EGC) is poor, with an overall 5-year survival rate of 10% worldwide [1]. In patients with metastatic EGC, curative treatment is no longer possible, resulting in an even worse prognosis with a median survival of less than a year [2–4]. Although EGC are two distinct entities, patients with metastatic adenocarcinoma of the esophagus and stomach are often studied collectively, because the dysregulation of oncogenic pathways and standard treatment often overlap [5]. Currently, chemotherapy with a combination of capecitabine and oxaliplatin (CAPOX) is the standard treatment for metastatic EGC in many centers [6,7]. Although some patients with metastatic disease do benefit substantially from this palliative systemic treatment, others do not while still experiencing adverse effects [3].

Contrast enhanced computed tomography (CT), following RECIST criteria, is the current standard for initial staging and follow-up (treatment response) evaluation of metastatic EGC [8]. This approach is valuable to determine tumor location, location of distant metastases and to evaluate treatment response based on size and spread of the disease. However, these size criteria are rather crude and changes can become apparent slower than actual treatment effects [9,10]. Furthermore, RECIST based criteria cannot be used for treatment response prediction and for evaluation of individual lesions.

Intra tumor heterogeneity has already been associated with treatment resistance in EGC [11]. More advanced image processing algorithms enable the assessment of this tumor heterogeneity, by extracting quantitative imaging biomarkers from standard medical images (e.g. CT). The so-called radiomics approach [12], where a combination of advanced image filtering is applied to extract image features, has the potential to extract prognostic phenotypes of individual lesions, as demonstrated in multiple cancer types [13–15]. Radiomics image features include textural features, information on tumor shape and size, and statistics on spatial intensity distributions before and after applying different image filters.

In esophageal cancer patients treated with neo-adjuvant chemoradiotherapy, studies have shown that CT-based textural features correlate with pathological tumor stage, response to treatment and overall survival [16–18]. The correlation with survival is also observed in patients treated with neo-adjuvant chemotherapy [19]. For gastric cancer several studies have investigated the relation between CT texture based tumor characteristics and tumor stage and grade, histopathological features, and survival after surgical resection or chemotherapy treatment [20–26].

Next to intra-tumoral heterogeneity, metastatic sites are also prone to develop their own tumor phenotype [27]. Hence, treatment response can differ between the primary tumor and metastases and between individual metastatic sites [28]. Previous studies have demonstrated correlation of CT textural features with pathological features and clinical outcome in liver metastases [29–31] However, generally only a small selection of the liver metastases a patient presents with are investigated. Due to the variety in response between lesions, a lesion based identification and prediction of response could help to get a more comprehensive view on the patient status and treatment options when other treatment regimens, as for instance metastasectomy, are considered [32]. In this study we investigated if a CT radiomics approach can predict response of individual liver metastases of EGC in patients treated with chemotherapy.

## Materials & methods

This study has been performed and reported according to the TRIPOD statement for the reporting of multivariable prediction models (S1 File) [33].

### Patient selection

Patients were retrospectively selected from all patients treated for advanced EGC in our institute in the period Jan 2011—Jan 2015 based on the following criteria: treated with at least 3 cycles of capecitabine and oxaliplatin, at least one liver metastasis present on the pre-treatment CT scan and evaluation CT scan performed after three cycles of therapy. A treatment cycle comprised a 3 weekly schedule of oxaliplatin (i.v., 130 mg/m^2^) on day 1, with capecitabine (p.o., 2 times a day, 1000mg/m^2^) on day 1-14. All procedures followed were in accordance with the ethical standards of the responsible committee on human experimentation (institutional and national). All patient scanning was performed under standard clinical routine and retrospective, anonymized, use of the data without further need for informed consent was approved by the Institutional Review Board of the Academic Medical Center.

### CT data

CT data for all potential patients were retrieved from the image archiving system. Since patients often received their pre-treatment CT scan at the referring hospital, images were acquired on different scanners with different scan protocols. Post-treatment CT scans were all made in our center. For quality assurance, scans were selected based on DICOM header information only including scans that were acquired with at least 120 kVp, a slice thickness of < = 5 mm and intra vascular contrast. The delayed contrast phase was used for lesion delineation and further feature extraction. To reduce variability between the data, scans were resampled to 1x1x2 mm^3^ voxels using linear interpolation [34].

### Image analysis

All scans were reviewed by a radiologist with 11 years of experience in abdominal imaging (B.M.). Images from the pre-treatment scan and evaluation scan were reviewed side by side in Velocity (Varian Medical Systems, Inc, Palo Alto, CA, USA) and liver metastases were delineated for each axial image slice to form a 3D region of interest (ROI) comprising the complete lesion in both scans. Care was taken to annotate corresponding lesions identically in both the pre-treatment and evaluation scan if still present. When necessary, pre and post treatment scans were aligned manually to be viewed side by side.

Within all lesion ROIs, a total of 370 radiomics features were extracted using an in-house developed radiomics toolbox (supported by Oncoradiomics, https://www.oncoradiomics.com/) implemented in Matlab 2016a (Mathworks, Natick, MA, USA). These features were based on a previous analysis in which these features were stable in esophageal cancer [35]. The total feature space comprised 7 first-order grey-level statistics describing lesion intensity, 19 features describing shape and size, 65 textural features describing the spatial distribution of voxel intensities, and 279 textural and statistics features extracted after three-dimensional wavelet transformation of the images. These features were derived from the grey-level co-occurrence (GLCM) [36], grey-level distance-zone (GLDZM) [37], grey-level run-length (GLRLM) [38], grey-level size-zone (GLSZM) [39], neighboring grey-level dependence (NGLDM) [40], and neighborhood grey-tone difference (NGTDM) [41] matrices. A list of used features is available in the supplementary materials (S1 Table). Mathematical definitions of the features are available in the supplementary materials of Lambin et al. [42].

### Statistical analysis

Statistical analysis was performed in R-3.2.5 (R: A Language and Environment for Statistical Computing, 2016, Vienna, Austria) and Matlab 2016a (Mathworks, Natick, MA, USA).

Responding lesions (RL) were defined as having a volume decrease of more than 65% between the pre-treatment CT and the evaluation CT scan. The 65% cut off was chosen as it represent the same amount of tumor shrinkage as a linear decrease of 30%, defined in the RECIST criteria as partial response and has shown similar performance in describing treatment response in previous studies [43,44]. A model was trained to discriminate RL from the remaining lesions based on the pre-treatment CT radiomics features.

To further evaluate the data, a subset of the RL was selected with 100% decrease in volume. These complete remission lesions (CR) were no longer detectable on the evaluation CT scan. Another model was built to select solely these lesions based on the pre-treatment CT radiomics features.

Models were built based on a random forest (RF) approach using the ‘randomForest’ packages [45], using 150 trees and a node size of 1. To validate the performance of the models, leave one out cross validation was used. First, all lesions from one patient were removed from the complete data set to be used as validation set. Next, the RF-model was trained on the lesions of the remaining patients. Then, the trained model was used to predict the outcome of the lesions from the validation patient. This process was repeated for all patients individually. The predicted outcomes of the lesions from all patients were combined to extract receiver operating characteristics (ROC) and plot the ROC curve of the model performance.

For all features the Pearson correlation coefficient with baseline lesion volume was calculated using the Matlab corrcoeff function, correlations with p<0.0001 were considered statistically significant.

The 10 most important features for both the RL and CR datasets were extracted based on the decrease in maximum Gini index averaged over the trained models.

## Results

### Patients & lesions

**Fig 1:**
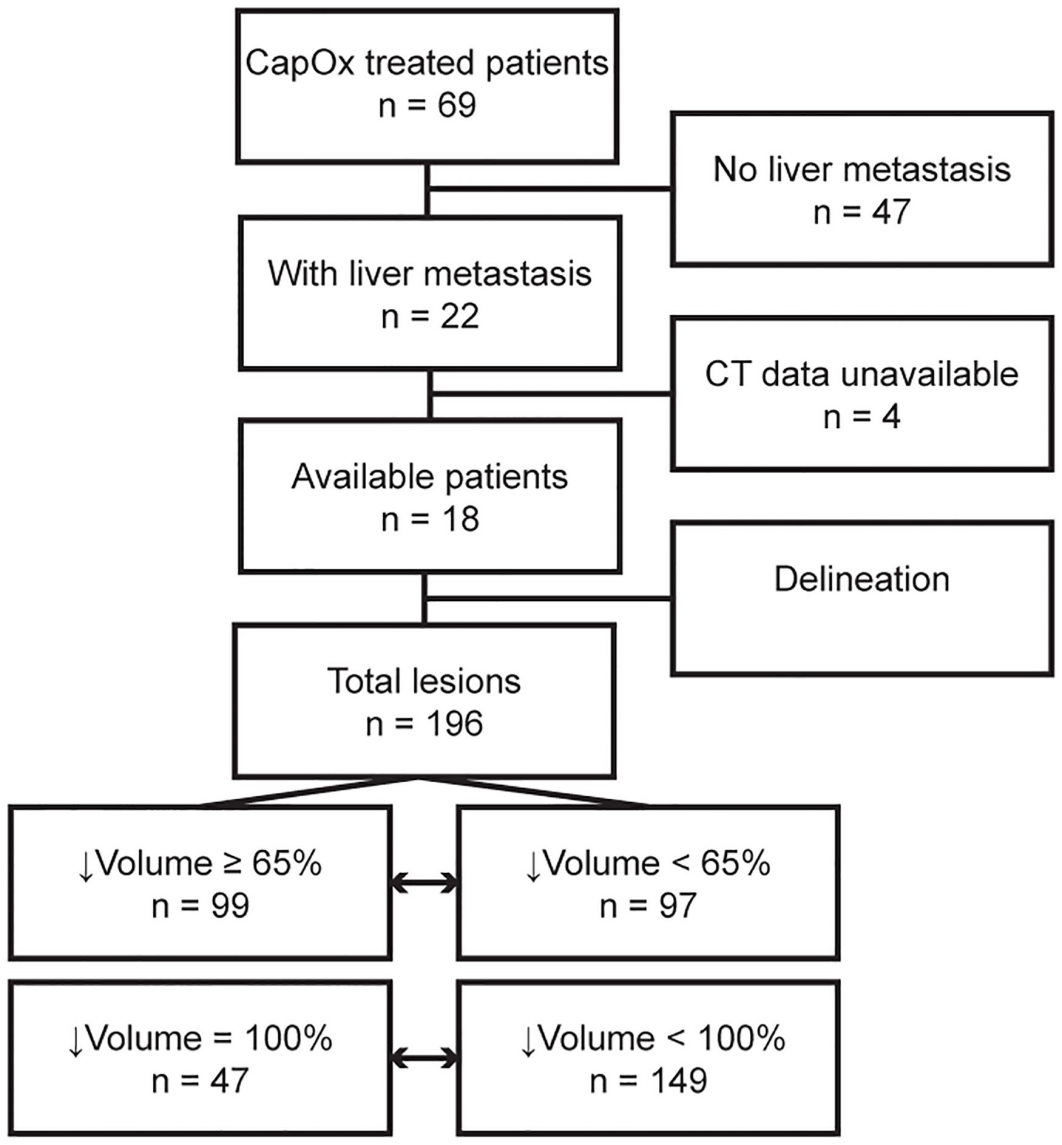
Flowchart of included patients.

Of the 69 patient treated with CAPOX in the inclusion period, 22 had liver metastases prior to CAPOX treatment. Four of these patients were excluded because the CT data could not be retrieved from the imaging archives. The remaining 18 patients had a total of 196 liver lesions on the pre-treatment CT scans with a median number of 10 (range 1–42) lesions per patient (Fig 1). Baseline characteristics of the included patients are summarized in Table 1.

**Table 1:** Patient characteristics.

Characteristic	Value (range)
Age (Y)	61 (37–78)
Sex	17 M / 1 F
Primary tumor	
Esophagus	8
Gastroesophageal junction	9
Stomach	1
Adeno / Squamous	16 / 2
Number of liver lesions	10 (1–42)
Lesion size (cm^3^)	1.89 (0.06–194.66)
RECIST after 3 cycles	
Partial response	10
Stable	2
Progressive	4
Mixed response	2
Median overall survival (M)	8.8 (4.2–24.1)

**Fig 2:**
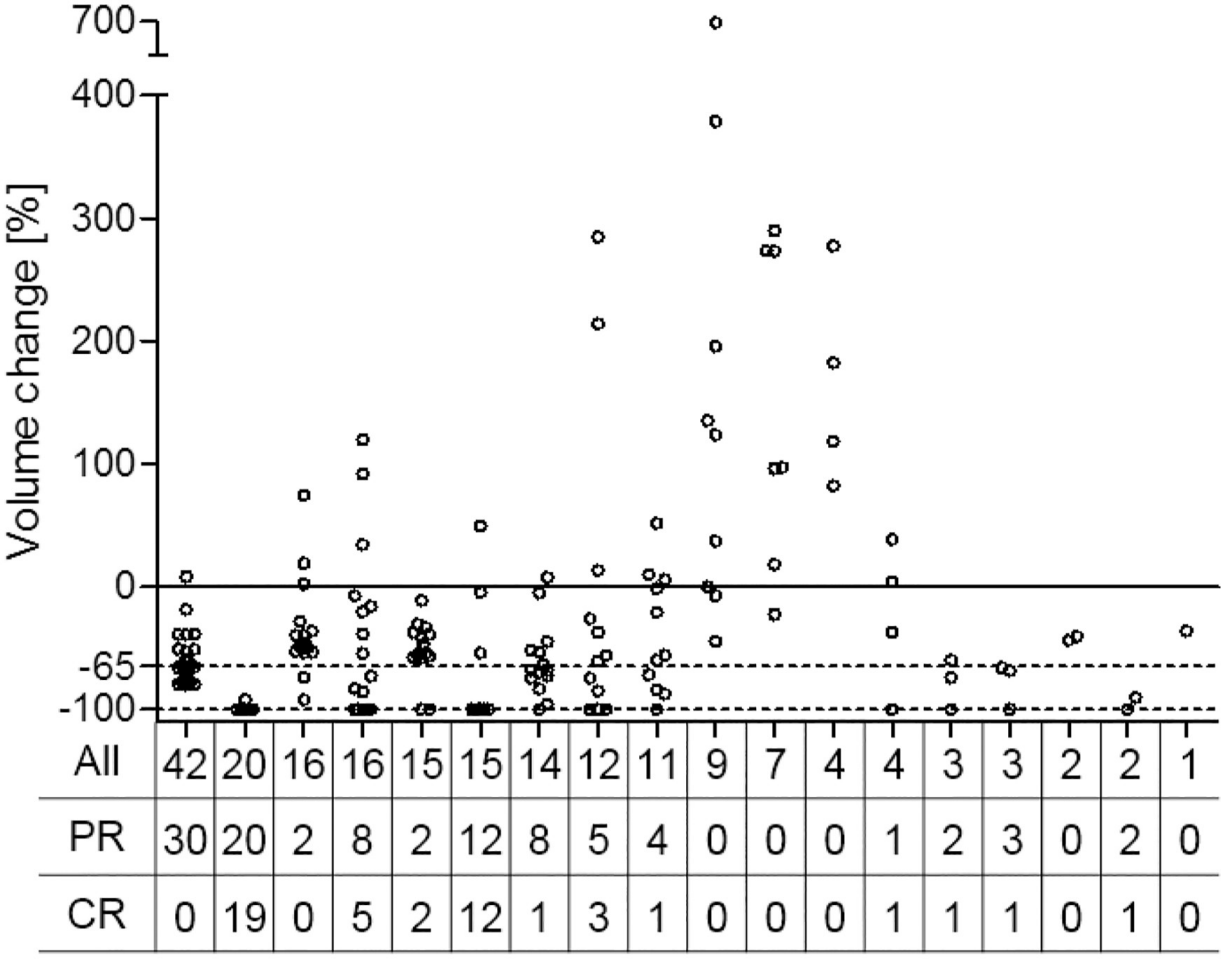
Proportional volume change of all liver metastases for each patient along with the total number of lesions (All), partial response (PR) and complete responding (CR) number of lesions. Note the spread of lesion volume change between and within individual patients.

Slightly more than half of the lesions, 99 (51%) in total, showed a decrease in size of more than 65% (RL set). The remaining 97 (49%) lesions showed a decrease in size of less than 65% or an increase in size. From the RL set 47 lesions (47% of RL, 24% of initial lesions) were no longer detected in scan 2 (CR set), compared to 149 (76% of initial) lesions that were still delineated on scan 2. Fig 2 illustrates the variation in lesion response amongst the included patients.

**Fig 3:**
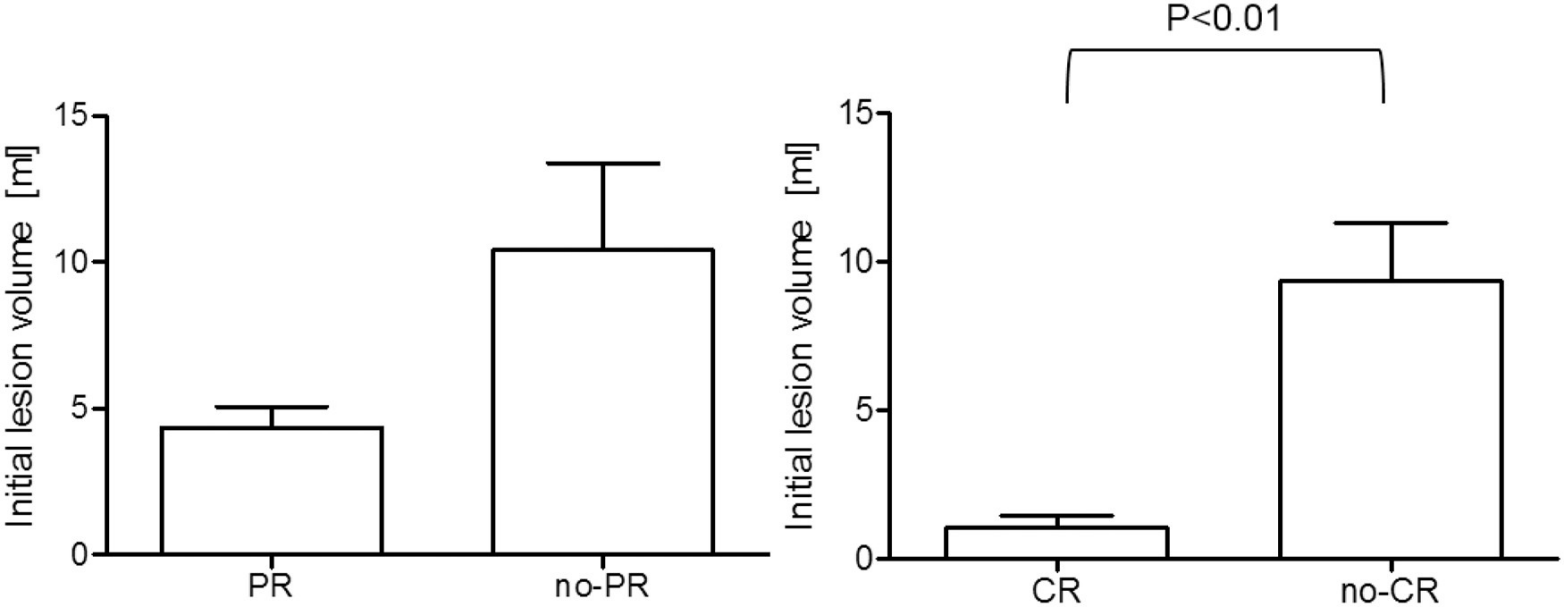
Median lesion size for the compared groups. CR lesions were significantly smaller at baseline than the other lesions.

Mean lesion volume in the pre-treatment CT of the RL set was 4.3 ± 7.3 vs. 10.4 ± 29.3 mL (p = 0.10) in the other lesions. The initial volume of the CR lesions (1.1 ± 2.6 mL) was significantly lower than the other lesions (9.3 ± 24.2 mL, p < 0.01) (Fig 3).

### Random forest model results

**Fig 4:**
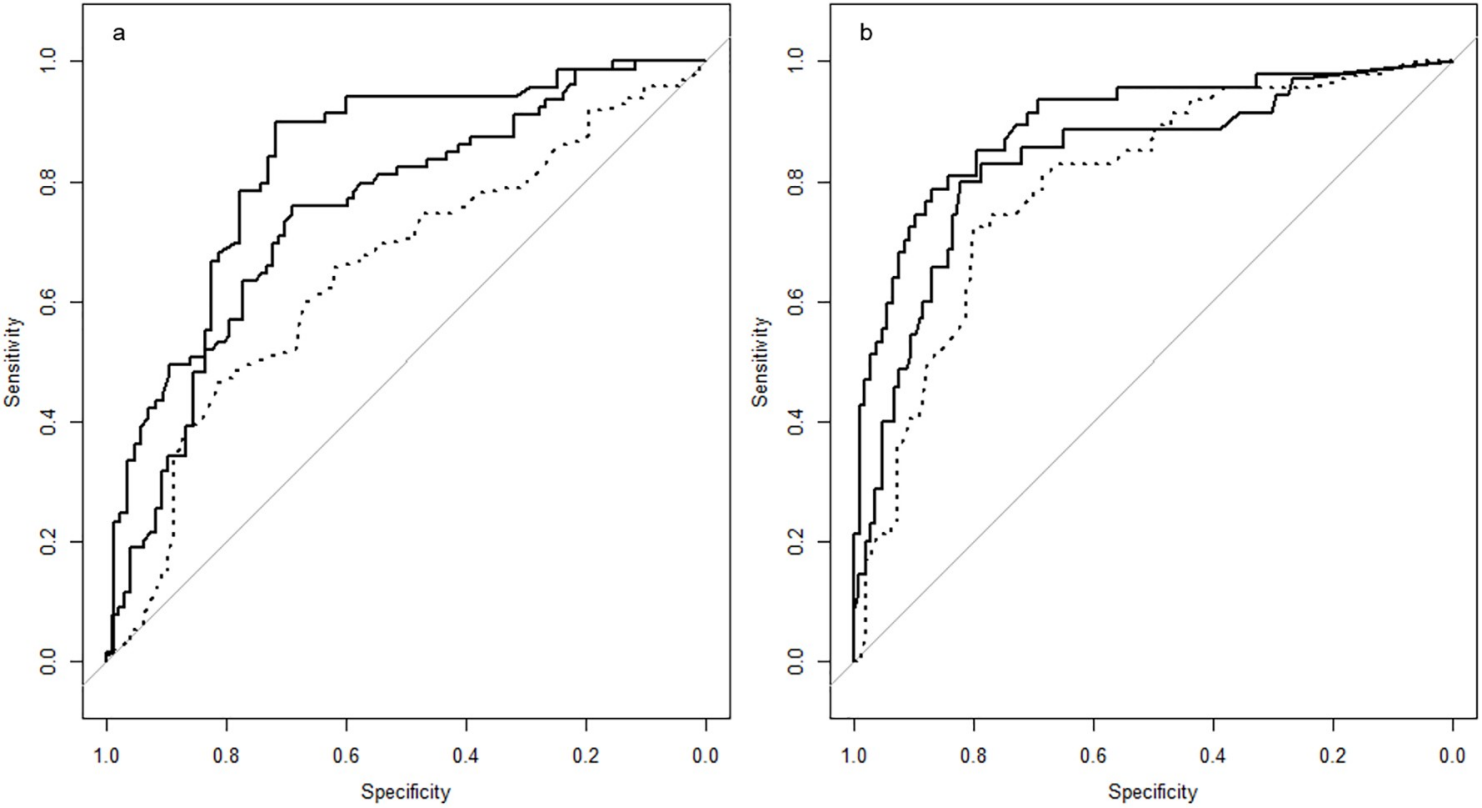
ROC curves of the PR (a) and CR (b) model for the best and worst training (solid) and the validation set (dashed).

The RF-model for the RL showed an average AUC of 0.79 (range: 0.74–0.83) for the 18 trained models. The ROC of the best and worst performing trained model are shown in Fig 4a along with the ROC of the combined validation set which had a AUC of 0.65 (95% ci 0.57–0.73).

For the CR set the RF-models performed better. The 18 trained models showed an average area under the curve of 0.87 (range: 0.83–0.90). The result of the best and worst performing training models are shown in Fig 4b along with the ROC curve of the combined validation sets which had an area under the curve of 0.79 (95% ci 0.72–0.87).

Performance plots for both models can be found in S1 and S2 Figs. The top 10 most important features are summarized in Table 2. Wavelet based features were dominant in discriminating lesions for both the RL and CR models. Furthermore, most of the features are a measure for tumor heterogeneity or describe the tumor intensity. Lesion volume on the pre-treatment CT scan did not show up in the top 10 features for both models. The correlation with initial lesion volume was limited and only significant in 2 out of the top 10 features for the RL model and one out of the top CR model features (Table 2). For the RL data the pre-treatment lesions volume alone reached an AUC of 0.35 (95% ci 0.27–0.43). For the CR data pre-treatment lesion volume alone was predictive with an AUC of 0.68 (95% ci 0.60–0.76).

**Table 2:** Top 10 important features according to the average decrease in Gini index over the 18 training models (features with a higher decrease tend to better split mixed nodes into single class nodes) and correlation coefficients of features with lesion volume.

RL Model			CR Model		
Radiomics Feature	Gini	r	Radiomics Feature	Gini	r
Wavelet_LHH_GLCM_correl1	1.44	0.06	Wavelet_HHH_GLDZM_LDE	1.15	0.22
GLCM_clusShade	1.24	0.00	Wavelet_HHH_GLDZM_SDE	1.00	0.30
Wavelet_LHH_Stats_rms	1.07	0.14	Wavelet_HHH_GLDZM_DZNN	1.00	-0.24
Wavelet_LLL_GLCM_clusShade	1.03	0.05	Wavelet_HHH_Stats_p10	0.84	0.05
Wavelet_LHH_Stats_std	1.03	-0.03	Wavelet_HHH_GLDZM_DZV	0.84	-0.17
Wavelet_LHH_Stats_p90	1.01	-0.06	Wavelet_HHL_Stats_p10	0.74	0.05
Wavelet_LHH_GLCM_infoCorr1	1.00	0.51*	GLSZM_ZP	0.70	-0.48*
Wavelet_LHH_Stats_var	0.91	-0.14	Wavelet_HHH_NGTDM_coarseness	0.67	-0.15
Wavelet_HHH_GLCM_infoCorr1	0.81	0.55*	Wavelet_HHH_Stats_iqr	0.67	-0.17
Wavelet_HHH_Stats_p10	0.73	0.05	Wavelet_HHL_Stats_iqr	0.66	-0.10

*p<0.0001

## Discussion

Here we studied treatment response prediction of liver metastases in patients with primary EGC based on radiomics features extracted from pre-treatment CT scans. To our knowledge we are the first to predict response of individual liver lesions to chemotherapy treatment. We found that radiomics features can predict a significant decrease in volume after therapy with an AUC of 0.64 (95% ci: 0.55–0.73) for PR and an 0.79 (95% ci 0.72–0.86) for CR lesions. This demonstrates that CT radiomics features are indeed sensitive to underlying tumor characteristics that influence therapy resistance in metastatic EGC.

As demonstrated in this and other studies, response can greatly vary within patients and is not completely described by standard used RECIST criteria. In our study patients showed a large variation in volume change after treatment, with some patients showing both evident volume decrease and increase. Although RECIST evaluation is not solely based on these liver lesions, this variation underlines the earlier findings that treatment response cannot be summarized with one label for all lesions in a patient [9,10]. Furthermore, RECIST evaluation is restricted to the choice of target lesions at the baseline scan, omitting changes in other lesions and limiting the evaluation as a whole. None the less, in our current approach we choose a RECIST associated cut off to identify responding lesions. Furthermore, variations in delineated volumes increase with decreasing lesion size, making reliable estimation of response more difficult [43]. Ideally, pathological response of each individual lesion would have been the reference standard as was recently done in gastric cancer [23]. However, pathological response is not evaluated as standard in this patient group with metastatic cancer. Furthermore, pathological treatment evaluation of all lesions would not be feasible in this respect with up to 42 individual lesions resulting in 42 percutaneous punctures per patient. Implications on how alternative response evaluation, whether or not based on size criteria, would influence our results should be investigated in dedicated, prospective, studies.

Although the AUC for the PR model was fairly limited, no alternatives are readily available. Existing models to predict patient outcome to chemotherapy in EGC mainly include clinical factors [46]. Although clinical factors could improve the performance of the current RF-models, it should be noted that clinical factors are patient based, so identical for all lesions of a patient, and therefore will not contribute to discriminate between lesions within a patient. In turn, adding CT based radiomics features to a clinical nomogram might improve prediction of patient outcome, since an independent measure of response is added to the data. The CR model performed better than the PR model. In a clinical context this might also be the most important model, since pre-treatment identification of CR lesions could aid in the further work-up of a patient to additional treatments. Identifying CR lesions implicitly also determines the non or partially responding lesions in a patient. Patients with only a small number of remaining non or partially responding lesions could potentially benefit most from additional, local, treatments which could then be targeted directly at the right lesions based on the model predictions [32]. Patients with no CR lesions could probably benefit most from a switch to alternative treatment regimens.

A large part of the lesions in our study only had limited volume at baseline. Although radiomics extraction from small lesions is known to be dependent on size [47], the features found most important in our models only showed limited correlation with lesion volume. This could partly be explained by the relatively high resolution of the CT images compared to for instance PET and that we resampled the images to 1x1x2mm^3^, therefore including more voxels in the same volume, making extraction of radiomics features more feasible. Furthermore, this suggest that more intrinsic, tumor phenotype based, features are dominant for predicting tumor response. Previous studies have also described the performance of CT textural features in primary esophageal and gastric cancer [16,17,19–26], showing promising results. Our study adds to the previous findings that, for esophageal cancer, most predictive markers are based on tumor heterogeneity. This heterogeneity might be attributed to tumor stroma, since this involves a complicated interaction of supporting cells, collagen and vascular networks, resulting in micro-structural differences between tumors. The tumor stroma has shown to play a decisive role in the development of treatment resistance in, amongst others, gastric cancer [11]. However, more direct comparisons between tissue characteristics and radiomics features, as were reported for lung cancer [48], are needed to further elucidate the underlying mechanism of radiomics features in esophageal cancer.

Patients with advanced EGC often present with multiple metastatic sites, so including other metastatic sites than the liver only. Main reason to focus on liver metastases was the high prevalence of liver metastases in this disease and the ability to reliably detect and delineate liver lesions in the CT data. Adding the primary lesion to the analysis might contribute to get a more complete prediction of patient response to treatment. However, since not all patients in our study had their primary tumor in situ, we chose not to include the primary tumor site in the current analysis. For this study, all individual liver lesions were delineated in each patient, up to 42 lesions in one patient. For future implementation this approach is far too time consuming. Furthermore, manual delineation is known to be prone to fluctuations that can also influence radiomics features [49]. More robust methods on lesion detection and automated segmentation algorithms have to be implemented to make this approach feasible in clinical routine, and have shown to improve repeatability of radiomics feature extraction [50].

The current study approach has its limitations. Although the total number of lesions was large for the current analysis and the lesion response showed a homogeneous distribution between patients, the total number of patients was small. Since some data dated back to as early as 2011 (start of CAPOX treatment in our center) we were not able to retrieve all data from the archiving systems due to technical issues introduced by data transitions and software changes. In a prospective setting this would not be an issue, since data would be readily available. We therefore don’t believe this affects generalizability of our study. Due to the small number of patients we did not perform survival analysis on patient basis. Inclusion of more patients will further improve the ability to generalize the current approach and to explore the possibilities of defining a predictive model for per patient survival prediction. Additionally, an independent dataset would help to further validate the result found in this study. The pre-treatment CT scans were performed in different institutes, meaning that different scan equipment and protocols were used. Radiomics features are known to be rather instable and differ between scan moments and parameters [34,35,51,52]. Although we did only use previously described stable features [35] and resampled the scans to an equal voxel size, instability of features could have influenced our findings in multiple ways. First, different scan parameters introduce scan specific variation in the data, making extraction of generic features from the overall data more challenging. Also, one patient with a lot of similar behaving lesions could also introduce a feature solely based on scan parameter fluctuation rather than intrinsic value. However, in daily clinical practice patients are often referred to different hospitals, while CT-scans have already been performed. Therefore our results, based on a heterogeneous dataset, might be more generalizable.

## Conclusion

Individual response of liver metastases varies greatly within and between patients. In this study, we demonstrated that a CT radiomics based approach shows potential in discriminating responding from non-responding liver metastases based on the pre-treatment CT scan. These results demonstrate the potential value of a CT radiomics approach in oncological practice of advanced EGC, although further validation in an independent patient cohort is needed to validate these findings.

## Supporting information

S1 Fig Accuracy plots of RL model. (TIF)

S2 Fig Accuracy plots of CR model. (TIF)

S1 Table Radiomics features included in the models. (DOCX)

S1 File Tripod checklist prediction model development. (PDF)

S2 File Supplementary data. (XLSX)

## References

[1] Jemal A, Bray F, Center MM, Ferlay J, Ward E, Forman D. Global cancer statistics. CA Cancer J Clin. 2011;61: 69–90. 10.3322/caac.20107 21296855 10.3322/caac.20107

[2] Bang Y-J, Van Cutsem E, Feyereislova A, Chung HC, Shen L, Sawaki A, et al Trastuzumab in combination with chemotherapy versus chemotherapy alone for treatment of HER2-positive advanced gastric or gastro-oesophageal junction cancer (ToGA): a phase 3, open-label, randomised controlled trial. Lancet. 2010;376: 687–697. 10.1016/S0140-6736(10)61121-X 20728210 10.1016/S0140-6736(10)61121-X

[3] Cunningham D, Starling N, Rao S, Iveson T, Nicolson M, Coxon F, et al Capecitabine and Oxaliplatin for Advanced Esophagogastric Cancer. N Engl J Med. 2008;358: 36–46. 10.1056/NEJMoa073149 18172173 10.1056/NEJMoa073149

[4] Wilke H, Muro K, Van Cutsem E, Oh S-C, Bodoky G, Shimada Y, et al Ramucirumab plus paclitaxel versus placebo plus paclitaxel in patients with previously treated advanced gastric or gastro-oesophageal junction adenocarcinoma (RAINBOW): a double-blind, randomised phase 3 trial. Lancet Oncol. 2014;15: 1224–1235. 10.1016/S1470-2045(14)70420-6 25240821 10.1016/S1470-2045(14)70420-6

[5] Kordes S, Cats A, Meijer SL, van Laarhoven HWM. Targeted therapy for advanced esophagogastric adenocarcinoma. Crit Rev Oncol Hematol. Elsevier Ireland Ltd; 2014;90: 68–76. 10.1016/j.critrevonc.2013.10.004 24183912 10.1016/j.critrevonc.2013.10.004

[6] Wagner AD, Syn NL, Moehler M, Grothe W, Yong WP, Tai B-C, et al Chemotherapy for advanced gastric cancer. Cochrane Database Syst Rev. 2017; 10.1002/14651858.CD004064.pub4 28850174 10.1002/14651858.CD004064.pub4 PMC6483552

[7] Ter Veer E, Haj Mohammad N, van Valkenhoef G, Ngai LL, Mali RMA, Anderegg MC, et al The Efficacy and Safety of First-line Chemotherapy in Advanced Esophagogastric Cancer: A Network Meta-analysis. J Natl Cancer Inst. 2016;108 10.1093/jnci/djw166 27576566 10.1093/jnci/djw166

[8] Eisenhauer EA, Therasse P, Bogaerts J, Schwartz LH, Sargent D, Ford R, et al New response evaluation criteria in solid tumours: Revised RECIST guideline (version 1.1). Eur J Cancer. Elsevier Ltd; 2009;45: 228–247. 10.1016/j.ejca.2008.10.026 19097774 10.1016/j.ejca.2008.10.026

[9] Desar IME, van Herpen CML, van Laarhoven HWM, Barentsz JO, Oyen WJG, van der Graaf WTA. Beyond RECIST: Molecular and functional imaging techniques for evaluation of response to targeted therapy. Cancer Treat Rev. Elsevier Ltd; 2009;35: 309–321. 10.1016/j.ctrv.2008.12.001 19136215 10.1016/j.ctrv.2008.12.001

[10] Trillet-Lenoir V, Freyer G, Kaemmerlen P, Fond A, Pellet O, Lombard-Bohas C, et al Assessment of tumour response to chemotherapy for metastatic colorectal cancer: accuracy of the RECIST criteria. Br J Radiol. 2002;75: 903–908. 10.1259/bjr.75.899.750903 12466256 10.1259/bjr.75.899.750903

[11] Wu Y, Grabsch H, Ivanova T, Tan IB, Murray J, Ooi CH, et al Comprehensive genomic meta-analysis identifies intra-tumoural stroma as a predictor of survival in patients with gastric cancer. Gut. 2013;62: 1100–1111. 10.1136/gutjnl-2011-301373 22735568 10.1136/gutjnl-2011-301373

[12] Lambin P, Rios-Velazquez E, Leijenaar R, Carvalho S, van Stiphout RGPM, Granton P, et al Radiomics: Extracting more information from medical images using advanced feature analysis. Eur J Cancer. 2012;48: 441–446. 10.1016/j.ejca.2011.11.036 22257792 10.1016/j.ejca.2011.11.036 PMC4533986

[13] Aerts HJWL, Velazquez ER, Leijenaar RTH, Parmar C, Grossmann P, Cavalho S, et al Decoding tumour phenotype by noninvasive imaging using a quantitative radiomics approach. Nat Commun. 2014;5: 4006 10.1038/ncomms5006 24892406 10.1038/ncomms5006 PMC4059926

[14] Leijenaar RTH, Carvalho S, Hoebers FJP, Aerts HJWL, van Elmpt WJC, Huang SH, et al External validation of a prognostic CT-based radiomic signature in oropharyngeal squamous cell carcinoma. Acta Oncol (Madr). 2015;54: 1423–1429. 10.3109/0284186X.2015.1061214 26264429 10.3109/0284186X.2015.1061214

[15] Coroller TP, Grossmann P, Hou Y, Rios Velazquez E, Leijenaar RTH, Hermann G, et al CT-based radiomic signature predicts distant metastasis in lung adenocarcinoma. Radiother Oncol. Elsevier Ireland Ltd; 2015;114: 345–350. 10.1016/j.radonc.2015.02.015 25746350 10.1016/j.radonc.2015.02.015 PMC4400248

[16] Yip C, Landau D, Kozarski R, Ganeshan B, Thomas R, Michaelidou A, et al Primary Esophageal Cancer: Heterogeneity as Potential Prognostic Biomarker in Patients Treated with Definitive Chemotherapy and Radiation Therapy. Radiology. 2014;270: 141–148. 10.1148/radiol.13122869 23985274 10.1148/radiol.13122869

[17] Ganeshan B, Skogen K, Pressney I, Coutroubis D, Miles K. Tumour heterogeneity in oesophageal cancer assessed by CT texture analysis: Preliminary evidence of an association with tumour metabolism, stage, and survival. Clin Radiol. The Royal College of Radiologists; 2012;67: 157–164. 10.1016/j.crad.2011.08.012 21943720 10.1016/j.crad.2011.08.012

[18] van Rossum PSN, Xu C, Fried D V., Goense L, Court LE, Lin SH. The emerging field of radiomics in esophageal cancer: current evidence and future potential. Transl Cancer Res. 2016;5: 410–423. doi: 10.21037/tcr.2016.06.19 10.21037/tcr.2016.06.19 PMC6343849 30687593

[19] Yip C, Davnall F, Kozarski R, Landau DB, Cook GJR, Ross P, et al Assessment of changes in tumor heterogeneity following neoadjuvant chemotherapy in primary esophageal cancer. Dis Esophagus. 2015;28: 172–179. 10.1111/dote.12170 24460831 10.1111/dote.12170

[20] Liu S, Liu S, Ji C, Zheng H, Pan X, Zhang Y, et al Application of CT texture analysis in predicting histopathological characteristics of gastric cancers. Eur Radiol. European Radiology; 2017;27: 4951–4959. 10.1007/s00330-017-4881-1 28643092 10.1007/s00330-017-4881-1

[21] Kim HY, Kim YH, Yun G, Chang W, Lee YJ, Kim B. Could texture features from preoperative CT image be used for predicting occult peritoneal carcinomatosis in patients with advanced gastric cancer? Simpson AL, editor. PLoS One. 2018;13: e0194755 10.1371/journal.pone.0194755 29596522 10.1371/journal.pone.0194755 PMC5875782

[22] Yoon SH, Kim YH, Lee YJ, Park J, Kim JW, Lee HS, et al Tumor heterogeneity in human epidermal growth factor receptor 2 (HER2)-positive advanced gastric cancer assessed by CT texture analysis: Association with survival after trastuzumab treatment. PLoS One. 2016;11: 1–10. 10.1371/journal.pone.0161278 27517841 10.1371/journal.pone.0161278 PMC4982686

[23] Giganti F, Marra P, Ambrosi A, Salerno A, Antunes S, Chiari D, et al Pre-treatment MDCT-based texture analysis for therapy response prediction in gastric cancer: Comparison with tumour regression grade at final histology. Eur J Radiol. Elsevier Ireland Ltd; 2017;90: 129–137. 10.1016/j.ejrad.2017.02.043 28583623 10.1016/j.ejrad.2017.02.043

[24] Ba-Ssalamah A, Muin D, Schernthaner R, Kulinna-Cosentini C, Bastati N, Stift J, et al Texture-based classification of different gastric tumors at contrast-enhanced CT. Eur J Radiol. Elsevier Ireland Ltd; 2013;82: e537–e543. 10.1016/j.ejrad.2013.06.024 23910996 10.1016/j.ejrad.2013.06.024

[25] Liu S, Shi H, Ji C, Zheng H, Pan X, Guan W, et al Preoperative CT texture analysis of gastric cancer: correlations with postoperative TNM staging. Clin Radiol. 2018; 10.1016/j.crad.2018.03.005 29625746 10.1016/j.crad.2018.03.005

[26] Giganti F, Antunes S, Salerno A, Ambrosi A, Marra P, Nicoletti R, et al Gastric cancer: texture analysis from multidetector computed tomography as a potential preoperative prognostic biomarker. Eur Radiol. European Radiology; 2017;27: 1831–1839. 10.1007/s00330-016-4540-y 27553932 10.1007/s00330-016-4540-y

[27] Almendro V, Kim HJ, Cheng Y-K, Gonen M, Itzkovitz S, Argani P, et al Genetic and Phenotypic Diversity in Breast Tumor Metastases. Cancer Res. 2014;74: 1338–1348. 10.1158/0008-5472.CAN-13-2357-T 24448237 10.1158/0008-5472.CAN-13-2357-T PMC3963810

[28] van Kessel CS, Samim M, Koopman M, van den Bosch MAAJ, Borel Rinkes IHM, Punt CJA, et al Radiological heterogeneity in response to chemotherapy is associated with poor survival in patients with colorectal liver metastases. Eur J Cancer. Elsevier Ltd; 2013;49: 2486–2493. 10.1016/j.ejca.2013.03.027 23692811 10.1016/j.ejca.2013.03.027

[29] Lubner MG, Stabo N, Lubner SJ, del Rio AM, Song C, Halberg RB, et al CT textural analysis of hepatic metastatic colorectal cancer: pre-treatment tumor heterogeneity correlates with pathology and clinical outcomes. Abdom Imaging. Springer US; 2015;40: 2331–2337. 10.1007/s00261-015-0438-4 25968046 10.1007/s00261-015-0438-4

[30] Miles K a, Ganeshan B, Griffiths MR, Young RCD, Chatwin CR. Colorectal Cancer: Texture Analysis of Portal Phase Hepatic CT Images as a Potential Marker of Survival. Radiology. 2009;250: 444–452. 10.1148/radiol.2502071879 19164695 10.1148/radiol.2502071879

[31] Rao S-X, Lambregts DM, Schnerr RS, Beckers RC, Maas M, Albarello F, et al CT texture analysis in colorectal liver metastases: A better way than size and volume measurements to assess response to chemotherapy? United Eur Gastroenterol J. 2016;4: 257–263. 10.1177/2050640615601603 27087955 10.1177/2050640615601603 PMC4804371

[32] Huddy JR, Thomas RL, Worthington TR, Karanjia ND. Liver metastases from esophageal carcinoma: is there a role for surgical resection? Dis Esophagus. 2015;28: 483–487. 10.1111/dote.12233 24898890 10.1111/dote.12233

[33] Collins G., Reitsma H, Altman D. Reporting Guideline for Prediction Model Studies : TRIPOD T ransparent R eporting of a multivariable prediction model for I ndividual P rognosis O r D iagnosis. Ann Intern Med. 2015;162: 55–63. 10.7326/M14-0697 25560714 10.7326/M14-0697

[34] Larue RTHM, van Timmeren JE, de Jong EEC, Feliciani G, Leijenaar RTH, Schreurs WMJ, et al Influence of gray level discretization on radiomic feature stability for different CT scanners, tube currents and slice thicknesses: a comprehensive phantom study. Acta Oncol (Madr). 2017; 1–10. 10.1080/0284186X.2017.1351624 28885084 10.1080/0284186X.2017.1351624

[35] Larue RTHM, Van De Voorde L, van Timmeren JE, Leijenaar RTH, Berbée M, Sosef MN, et al 4DCT imaging to assess radiomics feature stability: An investigation for thoracic cancers. Radiother Oncol. 2017;125: 147–153. 10.1016/j.radonc.2017.07.023 28797700 10.1016/j.radonc.2017.07.023

[36] Haralick RM, Shanmugam K, Dinstein I. Textural Features for Image Classification. IEEE Trans Syst Man Cybern. 1973;SMC-3: 610–621. 10.1109/TSMC.1973.4309314

[37] Thibault G, Angulo J, Meyer F. Advanced Statistical Matrices for Texture Characterization: Application to Cell Classification. IEEE Trans Biomed Eng. 2014;61: 630–637. 10.1109/TBME.2013.2284600 24108747 10.1109/TBME.2013.2284600

[38] Galloway MM. Texture analysis using gray level run lengths. Comput Graph Image Process. 1975;4: 172–179. 10.1016/S0146-664X(75)80008-6

[39] Thibault G, Fertil B, Navarro C, Pereira S, Cau P, Levy N, et al Shape and texture indexes application to cell nuclei classification. Int J Pattern Recognit Artif Intell. 2013;27: 1357002 10.1142/S0218001413570024

[40] Sun C, Wee WG. Neighboring gray level dependence matrix for texture classification. Comput Vision, Graph Image Process. 1983;23: 341–352. 10.1016/0734-189X(83)90032-4

[41] Amadasun M, King R. Textural features corresponding to textural properties. IEEE Trans Syst Man Cybern. 1989;19: 1264–1273. 10.1109/21.44046

[42] Lambin P, Leijenaar RTH, Deist TM, Peerlings J, Soest J de Jong EEC. Radiomics: the bridge between medical imaging and personalized medicine. Nat Rev Clin Oncol. 2017;In press. 10.1038/nrclinonc.2017.141 28975929

[43] Sohaib SA, Turner B, Hanson JA, Farquharson M, Oliver RTD, Reznek RH. CT assessment of tumour response to treatment: Comparison of linear, cross-sectional and volumetric measures of tumour size. Br J Radiol. 2000;73: 1178–1184. 10.1259/bjr.73.875.11144795 11144795 10.1259/bjr.73.875.11144795

[44] Prasad SR, Jhaveri KS, Saini S, Hahn PF, Halpern EF, Sumner JE. CT Tumor Measurement for Therapeutic Response Assessment: Comparison of Unidimensional, Bidimensional, and Volumetric Techniques—Initial Observations. Radiology. 2002;225: 416–419. 10.1148/radiol.2252011604 12409574 10.1148/radiol.2252011604

[45] Liaw A, Wiener M. Classification and Regression by randomForest. R news. 2002;2: 18–22.

[46] Custodio A, Carmona-Bayonas A, Jiménez-Fonseca P, Sánchez ML, Viudez A, Hernández R, et al Nomogram-based prediction of survival in patients with advanced oesophagogastric adenocarcinoma receiving first-line chemotherapy: a multicenter prospective study in the era of trastuzumab. Br J Cancer. 2017;116: 1526–1535. 10.1038/bjc.2017.122 28463962 10.1038/bjc.2017.122 PMC5518851

[47] Hatt M, Majdoub M, Vallieres M, Tixier F, Le Rest CC, Groheux D, et al 18F-FDG PET Uptake Characterization Through Texture Analysis: Investigating the Complementary Nature of Heterogeneity and Functional Tumor Volume in a Multi-Cancer Site Patient Cohort. J Nucl Med. 2015;56: 38–44. 10.2967/jnumed.114.144055 25500829 10.2967/jnumed.114.144055

[48] Grossmann P, Stringfield O, El-Hachem N, Bui MM, Rios Velazquez E, Parmar C, et al Defining the biological basis of radiomic phenotypes in lung cancer. Elife. 2017;6: 1–22. 10.7554/eLife.23421 28731408 10.7554/eLife.23421 PMC5590809

[49] van Velden FHP, Kramer GM, Frings V, Nissen IA, Mulder ER, de Langen AJ, et al Repeatability of Radiomic Features in Non-Small-Cell Lung Cancer [18F]FDG-PET/CT Studies: Impact of Reconstruction and Delineation. Mol Imaging Biol. Molecular Imaging and Biology; 2016;18: 788–795. 10.1007/s11307-016-0940-2 26920355 10.1007/s11307-016-0940-2 PMC5010602

[50] Parmar C, Rios Velazquez E, Leijenaar R, Jermoumi M, Carvalho S, Mak RH, et al Robust Radiomics Feature Quantification Using Semiautomatic Volumetric Segmentation. PLoS One. 2014;9: e102107 10.1371/journal.pone.0102107 25025374 10.1371/journal.pone.0102107 PMC4098900

[51] Mackin D, Fave X, Zhang L, Fried D, Yang J, Taylor B, et al Measuring Computed Tomography Scanner Variability of Radiomics Features. Invest Radiol. 2015;50: 757–765. 10.1097/RLI.0000000000000180 26115366 10.1097/RLI.0000000000000180 PMC4598251

[52] Shafiq-ul-Hassan M, Zhang GG, Latifi K, Ullah G, Hunt DC, Balagurunathan Y, et al Intrinsic dependencies of CT radiomic features on voxel size and number of gray levels. Med Phys. 2017;44: 1050–1062. 10.1002/mp.12123 28112418 10.1002/mp.12123 PMC5462462

