# Supplementary figures and images for: Feasibility of CT radiomics to predict treatment response of individual liver metastases in esophagogastric cancer patients
Source: PLoS One. 2018 Nov 15;13(11):e0207362. doi: 10.1371/journal.pone.0207362 (PMC6237370; doi:10.1371/journal.pone.0207362)

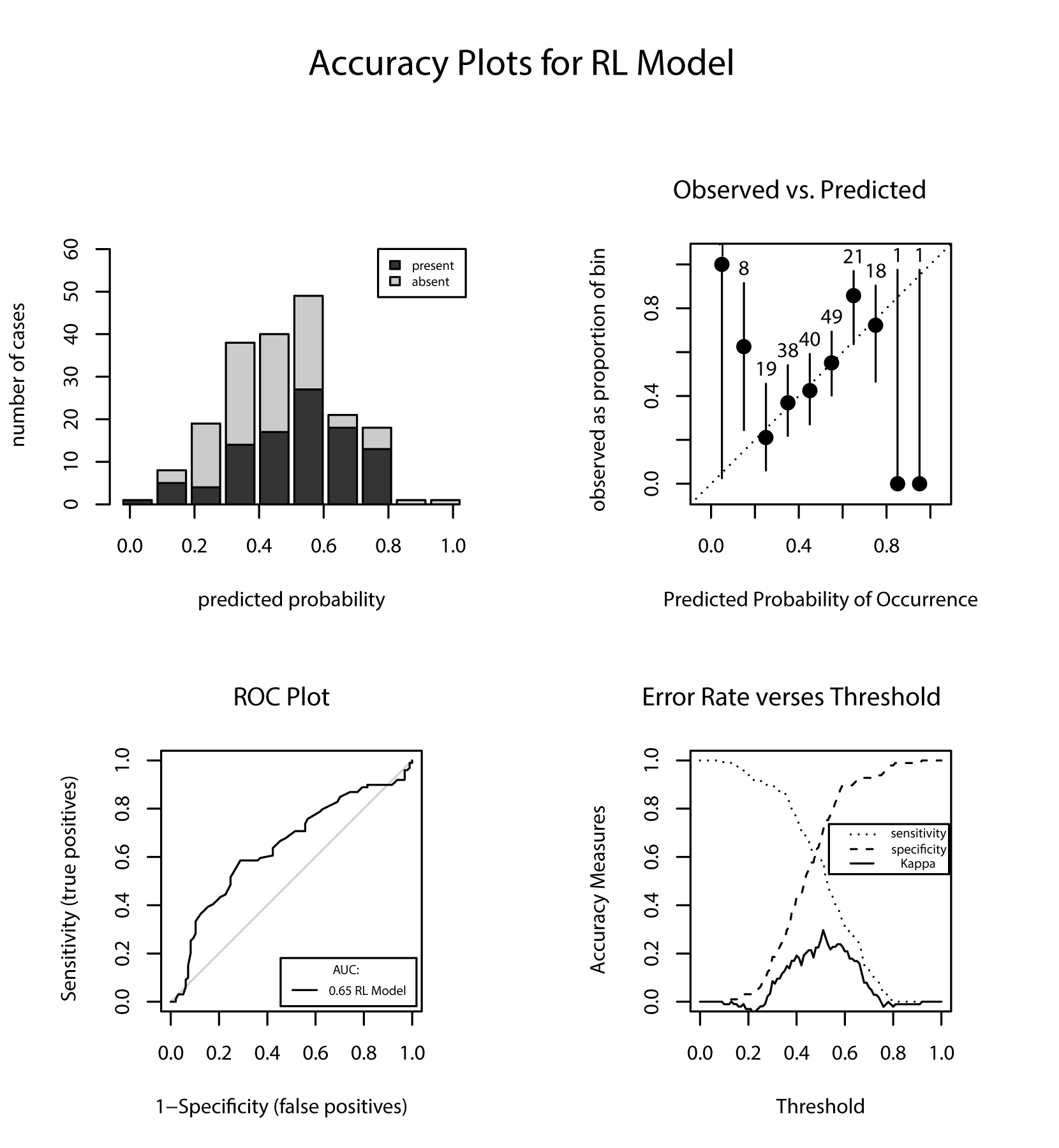

Supplement: S1 Fig — (TIF) [file pone.0207362.s001.tif]

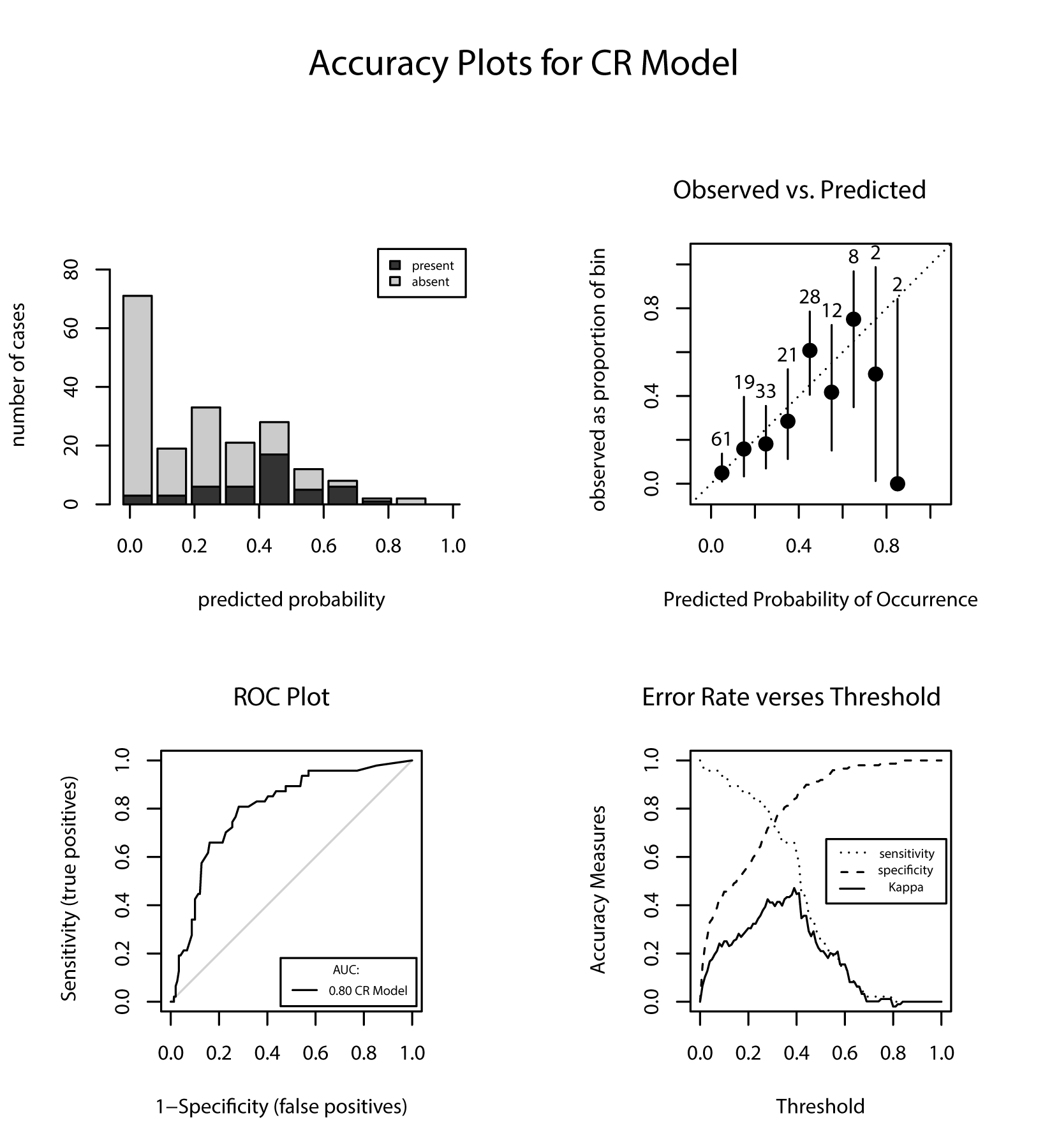

Supplement: S2 Fig — (TIF) [file pone.0207362.s002.tif]
