# Supplementary material for: Feasibility of CT radiomics to predict treatment response of individual liver metastases in esophagogastric cancer patients
Source: PLoS One. 2018 Nov 15;13(11):e0207362. doi: 10.1371/journal.pone.0207362 (PMC6237370; doi:10.1371/journal.pone.0207362)
Supplement: S1 Table — (DOCX) [file pone.0207362.s003.docx]

S1 Table Radiomics features included in the models.

| **'Feature Name'** |
| --- |
| 'GLCM_clusProm' |
| 'GLCM_clusShade' |
| 'GLCM_clusTend' |
| 'GLCM_contrast' |
| 'GLCM_diffAvrg' |
| 'GLCM_diffEntro' |
| 'GLCM_diffVar' |
| 'GLCM_dissimilar' |
| 'GLCM_energy' |
| 'GLCM_entrop2' |
| 'GLCM_homogeneity1' |
| 'GLCM_homogeneity2' |
| 'GLCM_infoCorr1' |
| 'GLCM_infoCorr2' |
| 'GLCM_invDiffnorm' |
| 'GLCM_inverseVar' |
| 'GLCM_maxProb' |
| 'GLCM_sumEntro' |
| 'GLCM_sumSquares' |
| 'GLCM_sumVar' |
| 'GLDZM_DZE' |
| 'GLDZM_DZN' |
| 'GLDZM_DZNN' |
| 'GLDZM_DZV' |
| 'GLDZM_HILDE' |
| 'GLDZM_IN' |
| 'GLDZM_INN' |
| 'GLDZM_IV' |
| 'GLDZM_LDE' |
| 'GLDZM_SDE' |
| 'GLDZM_ZP' |
| 'GLRLM_GLN' |
| 'GLRLM_GLNN' |
| 'GLRLM_GLV' |
| 'GLRLM_LRE' |
| 'GLRLM_LRHGE' |
| 'GLRLM_RE' |
| 'GLRLM_RLN' |
| 'GLRLM_RLNN' |
| 'GLRLM_RLV' |
| 'GLRLM_RP' |
| 'GLRLM_SRE' |
| 'GLSZM_HILAE' |
| 'GLSZM_IN' |
| 'GLSZM_INN' |
| 'GLSZM_IV' |
| 'GLSZM_LAE' |
| 'GLSZM_LILAE' |
| 'GLSZM_SZN' |
| 'GLSZM_SZV' |
| 'GLSZM_ZE' |
| 'GLSZM_ZP' |
| 'NGLDM_DN' |
| 'NGLDM_DNN' |
| 'NGLDM_DV' |
| 'NGLDM_GLN' |
| 'NGLDM_GLNN' |
| 'NGLDM_GLV' |
| 'NGLDM_HGLDE' |
| 'NGLDM_LDE' |
| 'NGLDM_SDE' |
| 'NGLDM_SM' |
| 'NGTDM_coarseness' |
| 'NGTDM_complexity' |
| 'NGTDM_strength' |
| 'Shape_asphericity' |
| 'Shape_centroidDistance' |
| 'Shape_compactness' |
| 'Shape_compactness2' |
| 'Shape_compactness3' |
| 'Shape_elongation' |
| 'Shape_flatness' |
| 'Shape_leastaxislength' |
| 'Shape_majoraxislength' |
| 'Shape_maxDiameter2D1' |
| 'Shape_maxDiameter2D2' |
| 'Shape_maxDiameter2D3' |
| 'Shape_maxDiameter3D' |
| 'Shape_minoraxislength' |
| 'Shape_spherDisprop' |
| 'Shape_sphericity' |
| 'Shape_surface' |
| 'Shape_surfVolRatio' |
| 'Shape_volume' |
| 'Stats_energy' |
| 'Stats_kurtosis' |
| 'Stats_max' |
| 'Stats_range' |
| 'Stats_skewness' |
| 'Stats_std' |
| 'Stats_var' |
| 'Wavelet_HHH_GLCM_average' |
| 'Wavelet_HHH_GLCM_infoCorr1' |
| 'Wavelet_HHH_GLCM_infoCorr2' |
| 'Wavelet_HHH_GLCM_sumAvg' |
| 'Wavelet_HHH_GLDZM_DZE' |
| 'Wavelet_HHH_GLDZM_DZN' |
| 'Wavelet_HHH_GLDZM_DZNN' |
| 'Wavelet_HHH_GLDZM_DZV' |
| 'Wavelet_HHH_GLDZM_HILDE' |
| 'Wavelet_HHH_GLDZM_IN' |
| 'Wavelet_HHH_GLDZM_LDE' |
| 'Wavelet_HHH_GLDZM_SDE' |
| 'Wavelet_HHH_GLDZM_ZP' |
| 'Wavelet_HHH_GLRLM_GLN' |
| 'Wavelet_HHH_GLRLM_RLN' |
| 'Wavelet_HHH_GLSZM_IN' |
| 'Wavelet_HHH_GLSZM_SZN' |
| 'Wavelet_HHH_NGLDM_DN' |
| 'Wavelet_HHH_NGLDM_GLN' |
| 'Wavelet_HHH_NGLDM_SM' |
| 'Wavelet_HHH_NGTDM_coarseness' |
| 'Wavelet_HHH_Stats_energy' |
| 'Wavelet_HHH_Stats_iqr' |
| 'Wavelet_HHH_Stats_p10' |
| 'Wavelet_HHH_Stats_p90' |
| 'Wavelet_HHH_Stats_rms' |
| 'Wavelet_HHH_Stats_std' |
| 'Wavelet_HHH_Stats_var' |
| 'Wavelet_HHL_GLCM_average' |
| 'Wavelet_HHL_GLCM_infoCorr1' |
| 'Wavelet_HHL_GLCM_infoCorr2' |
| 'Wavelet_HHL_GLCM_sumAvg' |
| 'Wavelet_HHL_GLDZM_DZE' |
| 'Wavelet_HHL_GLDZM_DZN' |
| 'Wavelet_HHL_GLDZM_DZNN' |
| 'Wavelet_HHL_GLDZM_DZV' |
| 'Wavelet_HHL_GLDZM_HILDE' |
| 'Wavelet_HHL_GLDZM_IN' |
| 'Wavelet_HHL_GLDZM_LDE' |
| 'Wavelet_HHL_GLDZM_SDE' |
| 'Wavelet_HHL_GLDZM_ZP' |
| 'Wavelet_HHL_GLRLM_GLN' |
| 'Wavelet_HHL_GLRLM_RLN' |
| 'Wavelet_HHL_GLSZM_IN' |
| 'Wavelet_HHL_GLSZM_SZN' |
| 'Wavelet_HHL_NGLDM_DN' |
| 'Wavelet_HHL_NGLDM_GLN' |
| 'Wavelet_HHL_NGLDM_SM' |
| 'Wavelet_HHL_NGTDM_coarseness' |
| 'Wavelet_HHL_NGTDM_complexity' |
| 'Wavelet_HHL_Stats_energy' |
| 'Wavelet_HHL_Stats_iqr' |
| 'Wavelet_HHL_Stats_p10' |
| 'Wavelet_HHL_Stats_p90' |
| 'Wavelet_HHL_Stats_rms' |
| 'Wavelet_HHL_Stats_std' |
| 'Wavelet_HLH_GLCM_average' |
| 'Wavelet_HLH_GLCM_infoCorr1' |
| 'Wavelet_HLH_GLCM_infoCorr2' |
| 'Wavelet_HLH_GLCM_invDiffnorm' |
| 'Wavelet_HLH_GLCM_sumAvg' |
| 'Wavelet_HLH_GLDZM_DZE' |
| 'Wavelet_HLH_GLDZM_DZN' |
| 'Wavelet_HLH_GLDZM_DZNN' |
| 'Wavelet_HLH_GLDZM_DZV' |
| 'Wavelet_HLH_GLDZM_HILDE' |
| 'Wavelet_HLH_GLDZM_IN' |
| 'Wavelet_HLH_GLDZM_LDE' |
| 'Wavelet_HLH_GLDZM_SDE' |
| 'Wavelet_HLH_GLDZM_ZP' |
| 'Wavelet_HLH_GLRLM_LRHGE' |
| 'Wavelet_HLH_GLRLM_RLN' |
| 'Wavelet_HLH_GLSZM_IN' |
| 'Wavelet_HLH_GLSZM_SZN' |
| 'Wavelet_HLH_NGLDM_DN' |
| 'Wavelet_HLH_NGLDM_SM' |
| 'Wavelet_HLH_NGTDM_coarseness' |
| 'Wavelet_HLH_NGTDM_complexity' |
| 'Wavelet_HLH_NGTDM_strength' |
| 'Wavelet_HLH_Stats_energy' |
| 'Wavelet_HLH_Stats_iqr' |
| 'Wavelet_HLH_Stats_kurtosis' |
| 'Wavelet_HLH_Stats_max' |
| 'Wavelet_HLH_Stats_min' |
| 'Wavelet_HLH_Stats_p10' |
| 'Wavelet_HLH_Stats_p90' |
| 'Wavelet_HLH_Stats_range' |
| 'Wavelet_HLH_Stats_rms' |
| 'Wavelet_HLH_Stats_std' |
| 'Wavelet_HLH_Stats_var' |
| 'Wavelet_HLL_GLCM_autocorr' |
| 'Wavelet_HLL_GLCM_average' |
| 'Wavelet_HLL_GLCM_clusShade' |
| 'Wavelet_HLL_GLCM_diffVar' |
| 'Wavelet_HLL_GLCM_infoCorr1' |
| 'Wavelet_HLL_GLCM_infoCorr2' |
| 'Wavelet_HLL_GLCM_invDiffmomnor' |
| 'Wavelet_HLL_GLCM_invDiffnorm' |
| 'Wavelet_HLL_GLCM_sumAvg' |
| 'Wavelet_HLL_GLDZM_DZE' |
| 'Wavelet_HLL_GLDZM_DZV' |
| 'Wavelet_HLL_GLDZM_HIE' |
| 'Wavelet_HLL_GLDZM_HISDE' |
| 'Wavelet_HLL_GLDZM_IN' |
| 'Wavelet_HLL_GLDZM_IV' |
| 'Wavelet_HLL_GLDZM_LDE' |
| 'Wavelet_HLL_GLDZM_ZP' |
| 'Wavelet_HLL_GLRLM_GLN' |
| 'Wavelet_HLL_GLRLM_GLV' |
| 'Wavelet_HLL_GLRLM_HGRE' |
| 'Wavelet_HLL_GLRLM_LRHGE' |
| 'Wavelet_HLL_GLRLM_RLN' |
| 'Wavelet_HLL_GLRLM_SRHGE' |
| 'Wavelet_HLL_GLSZM_HIE' |
| 'Wavelet_HLL_GLSZM_HISAE' |
| 'Wavelet_HLL_GLSZM_IN' |
| 'Wavelet_HLL_GLSZM_IV' |
| 'Wavelet_HLL_NGLDM_DN' |
| 'Wavelet_HLL_NGLDM_GLN' |
| 'Wavelet_HLL_NGLDM_HGE' |
| 'Wavelet_HLL_NGLDM_HGLDE' |
| 'Wavelet_HLL_NGLDM_HGSDE' |
| 'Wavelet_HLL_NGLDM_SM' |
| 'Wavelet_HLL_NGTDM_coarseness' |
| 'Wavelet_HLL_NGTDM_strength' |
| 'Wavelet_HLL_Stats_energy' |
| 'Wavelet_HLL_Stats_iqr' |
| 'Wavelet_HLL_Stats_min' |
| 'Wavelet_HLL_Stats_range' |
| 'Wavelet_HLL_Stats_rms' |
| 'Wavelet_HLL_Stats_std' |
| 'Wavelet_HLL_Stats_var' |
| 'Wavelet_LHH_GLCM_correl1' |
| 'Wavelet_LHH_GLCM_infoCorr1' |
| 'Wavelet_LHH_GLCM_infoCorr2' |
| 'Wavelet_LHH_GLDZM_DZE' |
| 'Wavelet_LHH_GLDZM_DZN' |
| 'Wavelet_LHH_GLDZM_DZNN' |
| 'Wavelet_LHH_GLDZM_DZV' |
| 'Wavelet_LHH_GLDZM_IN' |
| 'Wavelet_LHH_GLDZM_LDE' |
| 'Wavelet_LHH_GLDZM_SDE' |
| 'Wavelet_LHH_GLDZM_ZP' |
| 'Wavelet_LHH_GLRLM_GLN' |
| 'Wavelet_LHH_GLRLM_RLN' |
| 'Wavelet_LHH_GLSZM_IN' |
| 'Wavelet_LHH_GLSZM_SZN' |
| 'Wavelet_LHH_NGLDM_DN' |
| 'Wavelet_LHH_NGLDM_SM' |
| 'Wavelet_LHH_NGTDM_coarseness' |
| 'Wavelet_LHH_NGTDM_complexity' |
| 'Wavelet_LHH_Stats_energy' |
| 'Wavelet_LHH_Stats_iqr' |
| 'Wavelet_LHH_Stats_p10' |
| 'Wavelet_LHH_Stats_p90' |
| 'Wavelet_LHH_Stats_rms' |
| 'Wavelet_LHH_Stats_std' |
| 'Wavelet_LHH_Stats_var' |
| 'Wavelet_LHL_GLCM_average' |
| 'Wavelet_LHL_GLCM_correl1' |
| 'Wavelet_LHL_GLCM_infoCorr1' |
| 'Wavelet_LHL_GLCM_infoCorr2' |
| 'Wavelet_LHL_GLCM_invDiffmomnor' |
| 'Wavelet_LHL_GLCM_invDiffnorm' |
| 'Wavelet_LHL_GLCM_sumAvg' |
| 'Wavelet_LHL_GLDZM_DZN' |
| 'Wavelet_LHL_GLDZM_DZNN' |
| 'Wavelet_LHL_GLDZM_DZV' |
| 'Wavelet_LHL_GLDZM_IN' |
| 'Wavelet_LHL_GLDZM_LDE' |
| 'Wavelet_LHL_GLDZM_SDE' |
| 'Wavelet_LHL_GLDZM_ZP' |
| 'Wavelet_LHL_GLRLM_GLN' |
| 'Wavelet_LHL_GLRLM_RLN' |
| 'Wavelet_LHL_GLSZM_IN' |
| 'Wavelet_LHL_NGLDM_DN' |
| 'Wavelet_LHL_NGLDM_GLN' |
| 'Wavelet_LHL_NGLDM_SM' |
| 'Wavelet_LHL_NGTDM_coarseness' |
| 'Wavelet_LHL_NGTDM_complexity' |
| 'Wavelet_LHL_Stats_energy' |
| 'Wavelet_LHL_Stats_iqr' |
| 'Wavelet_LHL_Stats_max' |
| 'Wavelet_LHL_Stats_min' |
| 'Wavelet_LHL_Stats_p10' |
| 'Wavelet_LHL_Stats_p90' |
| 'Wavelet_LHL_Stats_range' |
| 'Wavelet_LHL_Stats_rms' |
| 'Wavelet_LHL_Stats_std' |
| 'Wavelet_LLH_GLCM_average' |
| 'Wavelet_LLH_GLCM_correl1' |
| 'Wavelet_LLH_GLCM_infoCorr1' |
| 'Wavelet_LLH_GLCM_infoCorr2' |
| 'Wavelet_LLH_GLCM_invDiffnorm' |
| 'Wavelet_LLH_GLCM_sumAvg' |
| 'Wavelet_LLH_GLDZM_DZE' |
| 'Wavelet_LLH_GLDZM_DZN' |
| 'Wavelet_LLH_GLDZM_DZNN' |
| 'Wavelet_LLH_GLDZM_DZV' |
| 'Wavelet_LLH_GLDZM_IN' |
| 'Wavelet_LLH_GLDZM_LDE' |
| 'Wavelet_LLH_GLDZM_SDE' |
| 'Wavelet_LLH_GLDZM_ZP' |
| 'Wavelet_LLH_GLRLM_GLN' |
| 'Wavelet_LLH_GLRLM_RLN' |
| 'Wavelet_LLH_GLSZM_IN' |
| 'Wavelet_LLH_GLSZM_SZN' |
| 'Wavelet_LLH_NGLDM_DN' |
| 'Wavelet_LLH_NGLDM_GLN' |
| 'Wavelet_LLH_NGLDM_SM' |
| 'Wavelet_LLH_NGTDM_coarseness' |
| 'Wavelet_LLH_Stats_energy' |
| 'Wavelet_LLH_Stats_iqr' |
| 'Wavelet_LLH_Stats_p10' |
| 'Wavelet_LLH_Stats_p90' |
| 'Wavelet_LLH_Stats_rms' |
| 'Wavelet_LLH_Stats_skewness' |
| 'Wavelet_LLH_Stats_std' |
| 'Wavelet_LLL_GLCM_clusShade' |
| 'Wavelet_LLL_GLCM_clusTend' |
| 'Wavelet_LLL_GLCM_contrast' |
| 'Wavelet_LLL_GLCM_diffAvrg' |
| 'Wavelet_LLL_GLCM_diffEntro' |
| 'Wavelet_LLL_GLCM_diffVar' |
| 'Wavelet_LLL_GLCM_dissimilar' |
| 'Wavelet_LLL_GLCM_energy' |
| 'Wavelet_LLL_GLCM_entrop2' |
| 'Wavelet_LLL_GLCM_homogeneity1' |
| 'Wavelet_LLL_GLCM_homogeneity2' |
| 'Wavelet_LLL_GLCM_infoCorr1' |
| 'Wavelet_LLL_GLCM_invDiffnorm' |
| 'Wavelet_LLL_GLCM_sumSquares' |
| 'Wavelet_LLL_GLCM_sumVar' |
| 'Wavelet_LLL_GLDZM_DZE' |
| 'Wavelet_LLL_GLDZM_DZN' |
| 'Wavelet_LLL_GLDZM_DZNN' |
| 'Wavelet_LLL_GLDZM_DZV' |
| 'Wavelet_LLL_GLDZM_HILDE' |
| 'Wavelet_LLL_GLDZM_IN' |
| 'Wavelet_LLL_GLDZM_IV' |
| 'Wavelet_LLL_GLDZM_LDE' |
| 'Wavelet_LLL_GLDZM_SDE' |
| 'Wavelet_LLL_GLDZM_ZP' |
| 'Wavelet_LLL_GLRLM_GLN' |
| 'Wavelet_LLL_GLRLM_GLV' |
| 'Wavelet_LLL_GLRLM_LRE' |
| 'Wavelet_LLL_GLRLM_LRHGE' |
| 'Wavelet_LLL_GLRLM_RE' |
| 'Wavelet_LLL_GLRLM_RLN' |
| 'Wavelet_LLL_GLRLM_RLNN' |
| 'Wavelet_LLL_GLRLM_RLV' |
| 'Wavelet_LLL_GLRLM_RP' |
| 'Wavelet_LLL_GLRLM_SRE' |
| 'Wavelet_LLL_GLSZM_HILAE' |
| 'Wavelet_LLL_GLSZM_IN' |
| 'Wavelet_LLL_GLSZM_IV' |
| 'Wavelet_LLL_GLSZM_LAE' |
| 'Wavelet_LLL_GLSZM_SZN' |
| 'Wavelet_LLL_GLSZM_SZV' |
| 'Wavelet_LLL_GLSZM_ZE' |
| 'Wavelet_LLL_GLSZM_ZP' |
| 'Wavelet_LLL_NGLDM_DN' |
| 'Wavelet_LLL_NGLDM_DNN' |
| 'Wavelet_LLL_NGLDM_DV' |
| 'Wavelet_LLL_NGLDM_GLN' |
| 'Wavelet_LLL_NGLDM_GLV' |
| 'Wavelet_LLL_NGLDM_HGLDE' |
| 'Wavelet_LLL_NGLDM_LDE' |
| 'Wavelet_LLL_NGLDM_SDE' |
| 'Wavelet_LLL_NGLDM_SM' |
| 'Wavelet_LLL_NGTDM_coarseness' |
| 'Wavelet_LLL_NGTDM_complexity' |
| 'Wavelet_LLL_NGTDM_strength' |
| 'Wavelet_LLL_Stats_energy' |
| 'Wavelet_LLL_Stats_max' |
| 'Wavelet_LLL_Stats_range' |
| 'Wavelet_LLL_Stats_skewness' |
| 'Wavelet_LLL_Stats_std' |
| 'Wavelet_LLL_Stats_var' |
